# Retrospective analysis of the role of cyclin E1 overexpression as a predictive marker for the efficacy of bevacizumab in platinum-sensitive recurrent ovarian cancer

**DOI:** 10.3332/ecancer.2021.1262

**Published:** 2021-07-05

**Authors:** Adriana Regina Gonçalves Ribeiro, Marcella Marineli Salvadori, Louise de Brot, Graziele Bovolin, Henrique Mantoan, Felipe Ilelis, Mariana Rezende, Nayra Soares do Amaral, Solange Moraes Sanches, Joyce Maria Lisboa Maya, Elizabeth Santana dos Santos, Ronaldo Pereira, Fabrício de Souza Castro, João Paulo da Nogueira Silveira Lima, Andrea Paiva Gadelha Guimarães, Glauco Baiocchi, Alexandre André Balieiro Anastácio da Costa

**Affiliations:** 1 Department of Medical Oncology, AC Camargo Cancer Center, 211 Professor Antonio Prudente Street, Liberdade, São Paulo, SP 01509-900, Brazil; 2 Department of Pathology, AC Camargo Cancer Center, 211 Professor Antonio Prudente Street, Liberdade, São Paulo, SP 01509-900, Brazil; 3 Department of Gynecologic Oncology, AC Camargo Cancer Center, 211 Professor Antonio Prudente Street, Liberdade, São Paulo, SP 01509-900, Brazil; a https://orcid.org/0000-0002-1631-0105

**Keywords:** bevacizumab, ovarian cancer, predictive markers, cyclin E1, platinum-free interval

## Abstract

The relative benefit of bevacizumab in ovarian cancer (OC) patients is greater the more the disease becomes platinum-resistant. Among other mechanisms of action, antiangiogenic agents may induce homologous recombination deficiency. Cyclin E1 (CCNE1) overexpression is a proposed marker of platinum resistance and is mutually exclusive with deficiency in homologous recombination. In this study, we evaluated the predictive value of CCNE1 expression with regard to the efficacy of bevacizumab. We retrospectively evaluated data from patients with platinum-sensitive recurrent OC who were treated with chemotherapy (CT) plus bevacizumab (Bev group) or CT alone (CT group) at a tertiary cancer centre from 2005 to 2017. The two groups were paired according to histology, platinum-free interval (PFI) and number of previous treatment lines. Progression-free survival (PFS) was compared between groups by log rank test and Cox regression. A total of 124 patients were included, with 62 in each group. The groups were well balanced regarding histology, PFI and number of previous treatment lines. Median PFS (mPFS) was 19.5 months for the Bev group versus 16.0 months for CT group (*p* = 0.150). By multivariate analysis, the HR for PFS was 2.25 (95% CI: 1.10–4.60) for CCNE1 overexpression. The benefit of bevacizumab was larger in the subgroups of patients with PFI 6–12 months (mPFS 18.6 versus 10.4 months, *p* = 0.002) and CCNE1 overexpression (mPFS 16.3 versus 7.0 months, *p* = 0.010). In conclusion, CCNE1 overexpression and PFI may suggest which patients will receive the greatest benefit from bevacizumab. These data, if confirmed by other studies, could help better select patients for antiangiogenic therapy.

## Background

Ovarian cancer (OC) is the second most common gynaecological malignancy and the most frequent cause of death from gynaecological cancer in the US, where 22,530 new cases and 13,980 deaths due to OC were estimated to have occurred in 2019 [1]. Worldwide, there are 295,414 new cases of OC and 184,799 deaths due to OC per year [2]. Despite initial therapy, 85% of patients will relapse during the first 4 years and require other treatments [3]. The backbone of systemic therapy remains platinum-based chemotherapy (CT), which can be followed by targeted maintenance therapies, including the antiangiogenic agent bevacizumab and oral poly ADP-ribose polymerase (PARP) inhibitors, which have been approved for the treatment of OC.

Bevacizumab is a humanised monoclonal antibody that is directed against vascular endothelial growth factor. The addition of bevacizumab to CT prolongs progression-free survival (PFS) in OC treatment, including first-line treatment [4, 5], treatment of recurrent OC in platinum-sensitive patients [6, 7] and in platinum-resistant patients [8]. Despite the gains derived from bevacizumab, the clinical value of this benefit remains disputed, because the absolute increase in disease control is on the order of few months, and no definitive improvement in overall survival (OS) has been seen in unselected populations.

A retrospective analysis of the two phase 3 first-line trials evaluated predictive biomarkers of the response to bevacizumab. The Gynecologic Oncology Group Study 0218 (GOG-0218) study examined tumour vascular endothelial growth factor receptor 2 (VEGFR2) expression and higher micro-vessel density, as measured by cluster of differentiation 31 (CD31) expression, and demonstrated their predictive value for PFS, but both markers failed after a longer follow-up [9]. The ICON7 study showed that among the four gene expression subtypes of OC, bevacizumab had the greatest benefit in the mesenchymal and proliferative subgroups [10]. These markers have not been validated and remain far from clinical practice. In the absence of antiangiogenic markers, intensification of therapy is usually prescribed to higher-risk patients.

Cyclin E1 (*CCNE1*) amplification is present in up to 20% of all high-grade serous carcinomas (HGSCs) and is associated with primary treatment resistance and lower OS in HGSC [11–14]. CCNE1 overexpression promotes unscheduled S-phase entry, disrupted DNA replication and genomic instability [15], necessitating intact homologous recombination repair pathways. Etemadmoghadam *et al* [16] reported the mutual exclusivity of homologous recombination pathway dysfunction and *CCNE1* amplification, which might explain the inadequate response to platinum therapy and thus the poor prognosis of CCNE1-overexpressing tumours. Antiangiogenic therapies induce a hypoxic cellular state that can downregulate homologous recombination-related genes (*BRCA1*, *BRCA2* and *RAD51*) [17].

Notwithstanding the lack of validated markers of the benefit of bevacizumab, patients with a shorter platinum-free interval (PFI) might derive greater benefit from bevacizumab, as evidenced by their lower hazard ratios for antiangiogenic therapy in the platinum-resistant scenario [8], and in a subgroup analysis of platinum-sensitive subjects, patients with a PFI of between 6 and 12 months received a greater benefit from bevacizumab compared with those with a PFI of longer than 12 months [6, 7].

In this study, we determined the benefit of bevacizumab in platinum-sensitive recurrent OC and calculated the predictive value of PFI and CCNE1 expression in the efficacy of bevacizumab.

## Methods

### Patients

**Figure 1.**
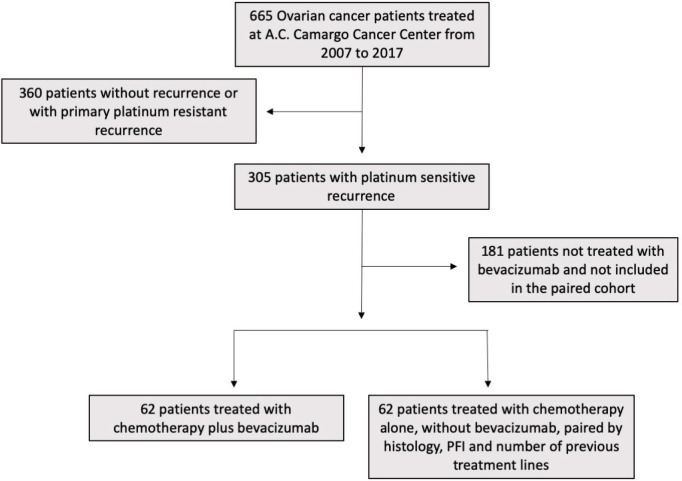
Flowchart representative of the inclusion criteria adopted in the study.

We retrospectively reviewed the medical charts of 124 patients who had received a diagnosis of ovarian carcinoma and were treated at AC Camargo Cancer Center (São Paulo, Brazil) between 2007 and 2017 (Figure 1). At this institution, patients are usually followed per local guidelines with medical visits every 3 months for cancer antigen 125 (CA125) measurements and contrast-enhanced abdominal and pelvic tomography during the first 2 years and imaging, CA125 and medical visits every 6 months for 5 years. All patients presented with platinum-sensitive recurrence, defined as a PFI of longer than 6 months. All consecutive patients who were treated with CT plus bevacizumab (Bev group) for platinum-sensitive disease were included. AC Camargo is a not-for-profit hospital that offers care for insured and uninsured patients (Brazilian public health system). Uninsured patients did not have access to bevacizumab, whereas this drug was offered to insured patients according to the discretion of the treating physician. Patients who were treated with CT alone (CT group), without bevacizumab, were paired 1:1 to patients in the Bev group. Pairing was done to keep the follow characteristics well balanced between the two groups: PFI, number of previous CT lines and histology. When exact matches for patients in the bevacizumab group were not found in the group of patients not treated with bevacizumab, matching factors were relaxed. This resulted in six cases (4.8%) not matched for PFI, five patients (3.1%) not matched for the number of previous treatment lines and two patients (1.2%) not matched for histology.

### Treatment

Recurrence of platinum-sensitive OC was treated per institutional protocols with one of the following options for an average of six cycles: carboplatin at an area under the curve (AUC) of 4 mg/mL/minute on Day 1 plus gemcitabine 1,000 mg/m^2^ on Days 1 and 8 every 21 days, carboplatin at an AUC of 4 mg/mL/minute on Day 1 plus doxorubicin 30 mg/m^2^ Day 1 every 28 days, carboplatin at an AUC of 5 mg/mL/minute on Day 1, and paclitaxel 175 mg/m^2^ on Day 1 every 21 days. Carboplatin can be exchanged for cisplatin 60 to 75 mg/m^2^ in cases of hypersensitivity. The dosage of bevacizumab was 15 mg/kg on Day 1 every 21 days, starting with CT and maintained as long as there was a benefit and no serious toxicities.

### Clinical data

The following clinical and pathological data was retrieved from the patients’ electronic records: age at the beginning of treatment, stage at diagnosis, tumour histology, family history of ovarian or breast cancer, personal history of breast cancer, residual disease after primary surgery, date of the last platinum infusion prior to recurrence, date of the platinum-sensitive recurrence by CA125 Gynecologic Cancer Intergroup (GCIG) criteria or Response Evaluation Criteria in Solid Tumors (RECIST) 1.1, CT for platinum-sensitive recurrence, use of bevacizumab, treatment with secondary cytoreduction, date of disease progression and date of last follow-up.

### Tissue samples

All available formalin-fixed, paraffin-embedded tumour tissue samples were used to construct a tissue microarray (TMA) after review by two dedicated gynaecologic oncology pathologists (LDB, GB).

## Immunohistochemistry

TMA sections were stained with primary antibodies against CCNE1 (HE12, sc-247 (Santa Cruz Biotechnology, Dallas, TX, USA); diluted 1:500). To validate the results with the antibody used in the TMA, we evaluated CCNE1 expression in whole sections of ten cases with a second antibody against CCNE1 (B7, sc-48420 (Santa Cruz Biotechnology, Dallas, TX, USA); diluted 1:50). The Polymer Detection System was used to detect the staining reactions (Novolink Max Polymer, Novocastra).

Immunohistochemical stains were analysed under a light microscope, and slides were interpreted by the two experienced gynaecological oncology pathologists, who were blinded to the clinical data. A histological score [Cyclin E (CICE)-score] was applied. Briefly, a score from 0 to 6 was calculated, based on nuclear staining intensity, ranging from 0 to 3, and the percentage of stained cells. The percentage of cells was scored 0 if no staining was observed, 1 if staining was seen in less than 50% of cells and 2 if staining was seen in over 50% of cells. We set the median score in the cohort as the cutoff for CCNE1 overexpression (CICE-score > 1).

### Statistical analysis

Frequencies were used to describe categorical variables. Median and interquartile range (IQR) values were used to describe continuous variables. Pearson’s chi-square test (or Fisher exact test when necessary) was used to compare baseline characteristics between the Bev and CT groups and to test the association between CCNE1 overexpression and clinical characteristics and between CCNE1 overexpression and objective response rate (ORR).

Correlation between CICE-score for CCNE1 expression evaluated with the two different antibodies was tested using Spearman’s coefficient and the concordance between the two different antibodies to determine CCNE1 overexpression was evaluated using kappa coefficient.

The response to treatment was recorded by the treating physician on medical charts as follows: ‘response’ if a complete or partial response was observed and ‘no response’ if stable disease or disease progression was seen.

For patients who were treated with bevacizumab for recurrent disease, PFS was calculated from the date of the first bevacizumab infusion until disease progression or death by any cause. Similarly, OS was calculated from the date of the first bevacizumab infusion until death by any cause. For patients who did not receive bevacizumab, their first or subsequent treatments for platinum-sensitive recurrences were analysed to maintain the same proportion of patients who were evaluated at the first-line treatment or at further treatment lines in parallel with the Bev group. PFS in the CT group was calculated from the date of the first CT infusion until disease progression or death by any cause, and OS was calculated from the date of the first CT infusion until death by any cause. Kaplan–Meier method was used to plot survival curves, and log-rank test was used to evaluate the impact of each variable on PFS and OS. As observed in other OC studies, nonproportional hazards were found for PFS (*p* = 0.041) according to bevacizumab treatment. To address this limitation, restricted mean survival times (RMSTs) were calculated and compared for PFS and OS over 20 and 40 months.

All clinical and pathological variables were tested by univariate analysis for PFS and OS using a Cox proportional hazards model. Variables with a *p*-value of less than 0.20 in the univariate analysis were selected for the multivariate analysis. Due to the limited number of events, the model was reduced by stepwise regression. The results were considered to be statistically significant when the *p*-value was less than 0.05. The statistical analysis was performed with SPSS (v.23, SPSS, Chicago, IL, USA).

The study conforms with The Code of Ethics of the World Medical Association (Declaration of Helsinki), printed in the British Medical Journal (18 July 1964). The study was approved by the ethics committee of AC Camargo Cancer Center (#2459/17).

## Results

The data for 124 patients with platinum-sensitive recurrent OC were retrospectively reviewed. Sixty-two (50%) patients were treated with CT plus bevacizumab, and 62 (50%) underwent CT alone without bevacizumab. The median age of the entire cohort was 57.5 years, and the most frequent histological type was HGSC (94.0% of cases). Seven patients presented other histological types: four patients presented high-grade endometrial carcinoma, one patient presented mucinous carcinoma and two patients presented low-grade serous carcinoma. Eighty-seven percent of patients presented with 2014 FIGO stage III–IV disease at the diagnosis, and 13% had FIGO stage I–II. Fifty-nine percent of patients received bevacizumab on the first relapse. The median PFI was 10.2 months. Secondary cytoreductive surgery (SCS) was performed in 62 (50%) patients. As described, the characteristics of the groups were well balanced in relation to PFI, histology and number of previous treatment lines. The groups were unbalanced regarding the CT regimen – in the Bev group, 52 (83.9%) patients were treated with a platinum-gemcitabine combination, whereas in the CT group, the most frequent treatment was a platinum-taxane combination, which was given to 30 (48.4%) patients (*p* < 0.001). The patient characteristics are summarised in Table 1.

**Figure 2.**
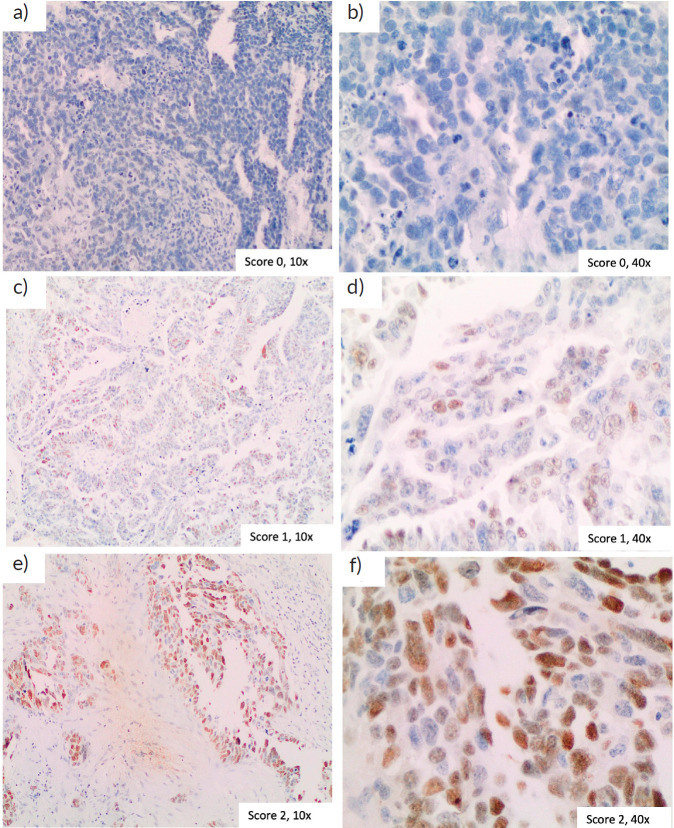
Immunohistochemistry of CCNE1. Examples of different final immunohistochemistry scores are shown. (a): Score 0, no positive cells, magnification 20×, (b): Score 1, magnification 20×, (c): Score 2, magnification 20×, (d) Score 4, magnification 20× and (e) Score 6, magnification 20×.

Among 124 patients, CCNE1 expression was assessed in 57 (Figure 2). We could not measure CCNE1 expression in the remaining tumours due to the unavailability of tumour samples. Nevertheless, the clinical features of CCNE1-assessed patients were consistent with those of the general cohort (Table 2).

CCNE1 overexpression was detected in 45.6% of tested patients – 15 (62.5%) in the Bev group and 11 (33.3%) in the non-Bev group (*p* = 0.029) (Table 1). Ten cases were used to validate TMA findings in whole tumour sections. One case had not enough tumour tissue. Among the remaining nine cases, there was a strong concordance (kappa = 0.780, *p* = 0.016) between the two antibodies in the classification of CCNE1 overexpression and a moderate correlation (Spearman’s coefficient = 0.682, *p* = 0.043) between the CICE-score of the two antibodies.

### Progression-free survival

**Figure 3.**
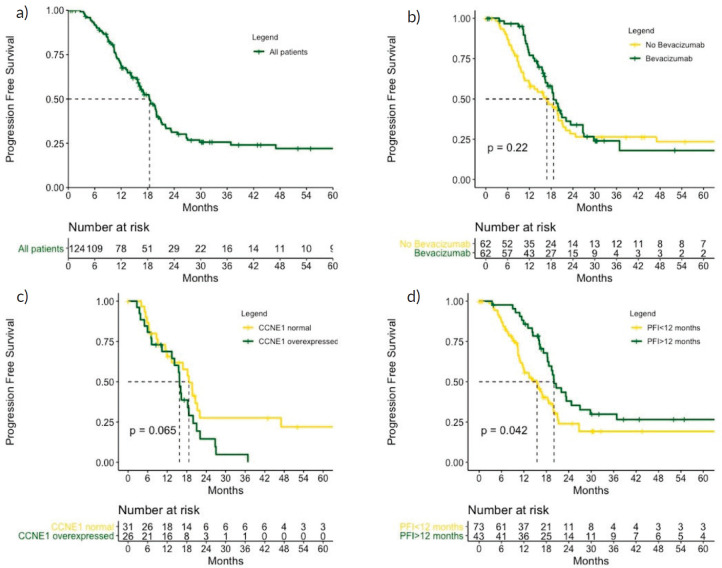
PFS (a): in the role cohort, (b): according to bevacizumab treatment, (c): according to CCNE1 expression and (d): according to PFI. CCNE1, Cyclin E1; PFI, Platinum free interval. p values calculated using log-rank test.

Disease progression was observed in 84 patients (67%), and the median PFS was 18.4 months. Patients who were treated with bevacizumab had a median PFS of 19.5 versus 16.0 months for those who did not receive bevacizumab (*p* = 0.220) (Figure 3). The RMST for PFS was 15.6 versus 13.4 months in favour of the Bev group (*p* = 0.006) after a follow-up of 18 months and 22.2 versus 19.8 months in favour of the Bev group (*p* = 0.310) after a follow-up of 40 months.

Patients with a PFI > 12 months had a median PFS of 20.0 versus 15.5 months for those with a PFI 6–12 months (*p* = 0.042). Patients who overexpressed CCNE1 had a median PFS of 15.8 months compared with 18.7 months for those with normal levels expression (*p* = 0.065) (Figure 3).

By univariate analysis, age < 65 years, high-grade serous histology, PFI > 12 months and CCNE1 overexpression were associated with PFS (Table 3) and were thus included in the multivariate analysis.

Bevacizumab treatment and CCNE1 overexpression remained in the final model, both of which were related to PFS (Table 4).

### Overall survival

**Figure 4.**
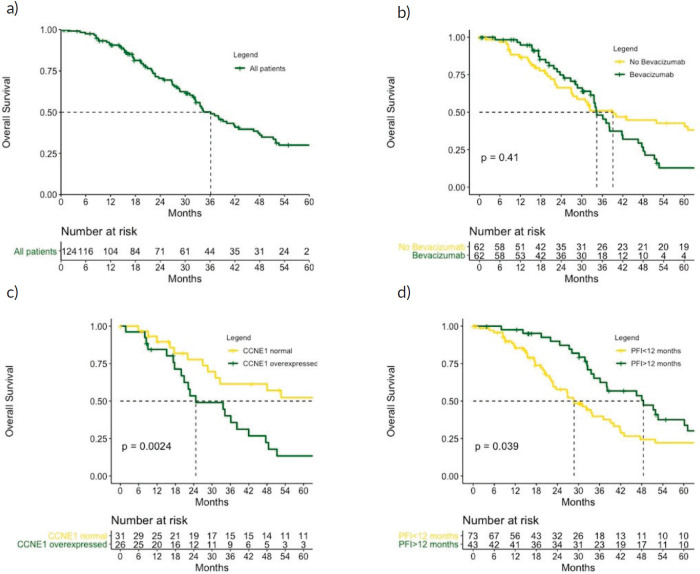
OS (a): in the role cohort, (b): according to bevacizumab treatment, (c): according to CCNE1 expression and (d): according to PFI. CCNE1, Cyclin E1; PFI, Platinum free interval. p values calculated using log-rank test.

After a median follow-up of 40.0 months, 61 patients had died (49.1%). The median OS was 51.3 months. Patients who were treated with bevacizumab had a median OS of 48.9 months compared with 51.9 months for those in the CT-only group (*p* = 0.410). PFI was a stronger predictor for OS: the median OS was 54.9 months for patients with PFI > 12 months versus 31.1 months for PFI 6–12 months (*p* = 0.039). Patients who overexpressed CCNE1 had a median OS of 24.7 versus 67.9 months for those with normal levels (*p* = 0.002) (Figure 4).

In the Cox univariate analysis, age < 65 years, high-grade serous histology, PFI 6–12 months, cytoreduction at diagnosis, family history of breast or OC and CCNE1 overexpression were associated with OS (Table 5).

When these factors were included in the multivariate analysis, only CCNE1 overexpression remained significantly associated with worse OS (Table 4).

### Objective response rate

The ORR for our cohort was 79.0%–93.3% in the Bev group and 67.9% in the CT group (*p* = 0.002). Patients with a PFI > 12 months had an ORR of 75.0% versus 84.2% for those with a PFI 6–12 months (*p* = 0.289). Patients with CCNE1 overexpression had an ORR of 75.0% compared with 70.8% for normal CCNE1 levels (*p* = 0.757).

### PFI and CCNE1 as predictive markers of the benefit of bevacizumab

**Figure 5.**
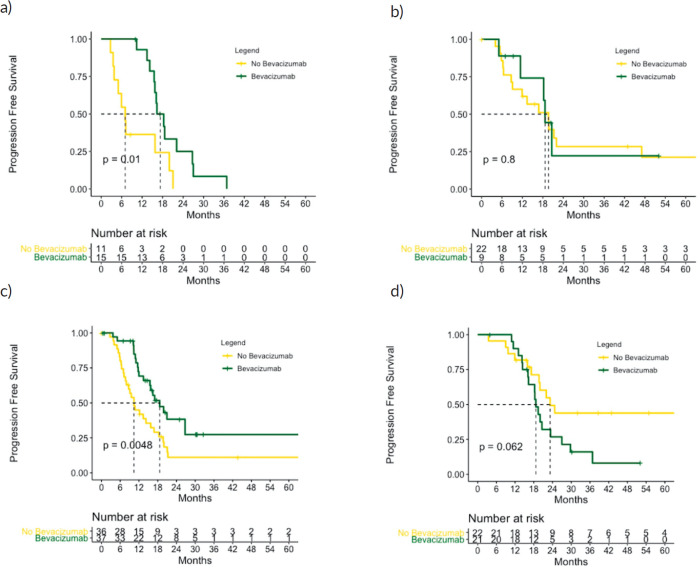
PFS according to bevacizumab treatment in the subgroups of patients with (a): CCNE1 overexpression, (b): CCNE1 normal, (c): PFI ≥ 12 months and (d): PFI 6–12 months. p values calculated using log-rank test.

**Figure 6.**
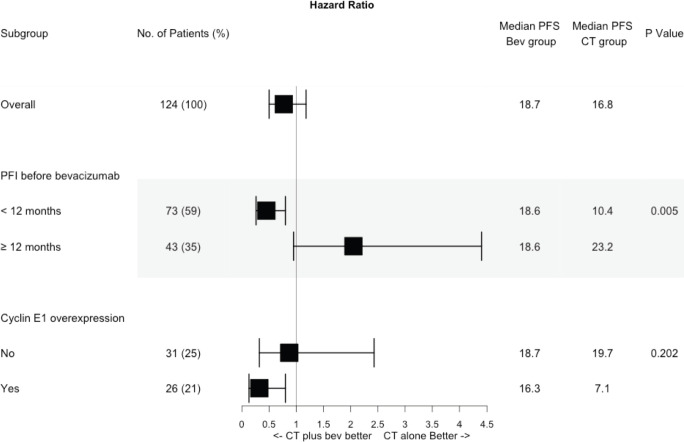
Forest plot showing hazard ratios from univariate cox regression for bevacizumab effect according to subgroups of different PFIs and CCNE1 expression. PFS, Progression free survival; PFI, Platinum free interval; Bev, Bevacizumab; CT, Chemotherapy.

The impact of bevacizumab differed by PFI and CCNE1 overexpression. The benefit of bevacizumab with regard to PFS was present only in the subgroups of patients with PFI 6–12 months (median PFS 18.6 versus 10.4 months, *p* = 0.005) and CCNE1 overexpression (median PFS 16.3 versus 7.1 months, *p* = 0.010) **–** not those with PFI > 12 months (median PFS 18.6 versus 23.2 months, *p* = 0.062) or normal CCNE1 expression (median PFS 19.7 versus 18.7 months, *p* = 0.796). By interaction test, the value for the effect of bevacizumab on PFI was 0.005 and 0.202 on CCNE1 (Figures 5 and 6). Among patients who overexpressed CCNE1, the ORR was 100% for the Bev group and 50% for the CT group (*p* = 0.033).

## Discussion

This retrospective analysis of platinum-sensitive recurrent OC implicates PFI and CCNE1 expression as markers of a benefit from bevacizumab. We confirmed the benefit of bevacizumab on PFS in the platinum-sensitive setting of recurrent OC and the prognostic value of PFI. CCNE1 overexpression was found to be a factor of a worse prognosis, and overexpressing patients derived the largest benefit from the introduction of bevacizumab, whereas low-expressing patients had no benefit.

Bevacizumab consistently improves PFS in all settings of OC treatment, during primary treatment [4, 5], platinum-sensitive recurrence [6, 7] and platinum-resistant recurrence [8], but lacks a predictive biomarker. In the first-line setting, microvascular density – as evaluated by CD31 expression – and proliferative and mesenchymal gene expression subtypes were found to be associated with the efficacy of bevacizumab in the GOG218 and International Collaborative Ovarian Neoplasm Trial 7 (ICON7) trials, respectively [9, 10]. Despite these initial findings, they have not been prospectively validated, and there is no well-established biomarker (predictive) of the benefit of bevacizumab.

The magnitude of the benefit from bevacizumab in our study (HR of 0.54 for PFS) is consistent with large prospective studies [6, 7]. Patients in the two groups were paired with regard to the main prognostic factors, such as PFI, tumour histology and number of previous treatment lines. No features were unbalanced between the Bev and CT groups, with the exception of CCNE1 overexpression, of which more of the former experienced. Notably, the treating physicians prescribed bevacizumab (or not) without previous knowledge of CCNE1 status.

The benefit from bevacizumab was present only in patients who overexpressed CCNE1. This gain can be explained by the platinum resistance in CCNE1-overexpressing tumours and not the direct interaction between antiangiogenic therapy and CCNE1 overexpression. Conversely, antiangiogenic therapy leads to a deficiency in homologous recombination [17], and *CCNE1* amplification is mutually exclusive to such a deficiency [18, 19] due to synthetic lethality between the two pathways [16]. This relationship could constitute the basis for the predictive value of CCNE1 overexpression in our study.

Due to the mutual exclusivity with homologous recombination deficiency, the predictive value could be driven by the disparate efficacies of antiangiogenic therapy between homologous recombination-deficient tumours. This topic is controversial in the literature. Whilst retrospective evaluation of the first-line bevacizumab trial from GOG did not find any association of homologous recombination-related gene mutations with the efficacy of bevacizumab [20], at least one study suggests that some patients with tumours with homologous recombination deficiency may not benefit from bevacizumab [21].

We found that CCNE1 overexpression was associated with shorter PFS and OS in the platinum-sensitive scenario. Previous studies have shown that *CCNE1* amplification is more frequent in primary platinum-resistant tumours [11, 12] and correlates with a worse prognosis after primary treatment [14, 18]. Although the evaluation of *CCNE1* amplification on the genomic level is more accurate as a prognostic marker [14], overexpression of the protein has also been related to the prognosis [13]. Our study is the first to show the prognostic value of CCNE1 overexpression in the platinum-sensitive setting, notably among patients with partially platinum-sensitive disease (PFI 6–12 months) that account for the majority of the patients in the study.

PFI is the most important prognostic factor in OC recurrence [22]. It is also a marker of the response to platinum re-exposure: a longer PFI is associated with a greater probability of a response to platinum therapy [23]. In the two phase 3 trials of bevacizumab in the platinum-sensitive scenario, patients with a PFI of between 6 and 12 months had a greater benefit from bevacizumab than those with a longer PFI [6, 7]. Moreover, among all trials in OC, the greatest relative benefit of bevacizumab was seen in the platinum-resistant trial [8]. In our study, the PFI affected the benefit from bevacizumab, which was significant in patients with a PFI of 6–12 months but non-significant in those with a PFI of longer than 12 months. The absence of a benefit in the longer-PFI subgroup in this study might have stemmed from small sample and inherent limited statistical power, although the statistically significant interaction test (*p* = 0.005) identified a distinct benefit from bevacizumab according to PFI.

The larger benefit of bevacizumab in borderline platinum-sensitive patients (PFI 6–12 months) might derive from lower platinum sensitivity in this scenario. With less effective CT, a constant effect of bevacizumab would result in a greater relative benefit. Notwithstanding this hypothesis, at least one study has suggested a more important function of angiogenesis in platinum-resistant tumours versus platinum-sensitive tumours, with higher expression of VEGFR2 in the former [24]. The biological background for this hypothesis should be investigated further.

As a clinical marker of the efficacy of bevacizumab, PFI could not be taken into account, because the results came from a subgroup analysis and because the benefit from bevacizumab was present in trials for entire population. But with today’s option of PARP inhibitors for maintenance treatment, even for patients with wild-type *BRCA* in the platinum-sensitive scenario [25–27], these data could help guide decisions on the best maintenance option for these patients.

In our study, nonproportional hazards for bevacizumab for PFS were present, as in several randomised bevacizumab trials [28]. Notably, the benefit of bevacizumab in CCNE1-overexpressing patients had a proportional hazards pattern.

Our study had several limitations due to its retrospective nature, the small number of patients – especially in the CCNE1 analysis, with a statistically insignificant interaction (*p* = 0.202) – , no adjustment for multiple comparisons and the absence of external validation, limiting any definitive conclusion. The small number of patients limits the conclusions about the subgroups in whom we did not see a clear benefit of bevacizumab use due to lack of statistical power. Inclusion of patients from both private and public health systems could potentially introduce a bias. Despite this, among 118 patients with available data about the payment source (private versus public health systems), there was no difference in PFS among the two groups, 18.7 versus 17.2 months (*p* = 0.338). Despite these limitations, the two treatment groups were paired for the most important prognostic factors, and the findings regarding CCNE1 overexpression as a predictive marker for bevacizumab had a strong biological background.

## Conclusions

In conclusion, our study confirms PFI as a predictive marker of the efficacy of bevacizumab and raises the hypothesis that CCNE1 may be both a marker of poor prognosis and predictive marker of the benefit of bevacizumab. If validated, these findings could lead to better selection of patients for antiangiogenic therapy and improved identification of CCNE1-overexpressing OCs as a different class of tumours that merit a particular treatment approach.

## List of abbreviations

AUC, Area under the curve; Bev, Bevacizumab; CCNE1, Cyclin E1; CT, Chemotherapy; HGSC, High-grade serous carcinoma; HR, hazard ratio; IQR, Interquartile range; OC, Ovarian cancer; ORR, Objective response rate; OS, Overall survival; PARP, Poly ADP-ribose polymerase; PFI, Platinum-free interval; PFS, Progression-free survival; RMST, Restricted mean survival time; TMA, Tissue micro-array

## Conflict of interests

The authors declare no conflict of interest related to this study.

## Figures and Tables

**Table 1.** Clinical characteristics of the 124 patients.

Characteristic	Freq. (%)		*p* value^a^
	Bevacizumab	No bevacizumab	
Number of patients	62	62	
Age, years			
Median (IQR)	56.2 (48.2–63.5)	59.2 (52.0–68.1)	0.091^b^
Stage			
I–II	6 (10.0)	9 (15.8)	0.349
III–IV	54 (90.0)	48 (84.2)	
Tumour histology			
High-grade serous	56 (94.9)	54 (93.1)	0.680
Others	3 (5.10)	4 (6.90)	
Family history of ovarian or breast cancer			
No	39 (69.6)	35 (62.5)	0.425
Yes	17 (30.4)	21 (37.5)	
Residual disease after primary surgery			
≤10 mm	45 (76.3)	35 (70.0)	0.460
>10 mm	14 (23.7)	15 (30.0)	
PFI before bevacizumab – Median (IQR)	9.7	10.5	0.383^b^
6–12 months	37 (63.8)	34 (60.7)	0.735
≥12 months	21 (36.2)	22 (39.3)	
CT regimen			
Platinum + taxane	9 (14.5)	30 (48.4)	<0.001
Platinum + gemcitabine	52 (83.9)	26 (41.9)	
Platinum + liposomal doxorubicin	0 (0.00)	5 (8.1)	
Monotherapy	1 (1.60)	1 (1.0)	
Number of previous treatment lines			
1	34 (55.7)	37 (61.7)	0.868
2	20 (32.8)	18 (30.0)	
3	5 (8.20)	3 (5.0)	
4	2 (3.30)	2 (3.3)	
Surgery			
Primary Cytoreduction	45 (76.3)	35 (70.0)	0.460
Interval cytoreduction	14 (23.7)	15 (30.0)	
SCS			
Yes	28 (62.2)	34 (61.8)	0.539
No	17 (37.8)	21 (38.2)	
CCNE1 overexpression			
Yes 19	15 (62.5)	11 (33.3)	0.029
No	9 (37.5)	22 (66.7)	

a All *p* values calculated by chi square test unless otherwise specified

b *p* value calculated with Mann–Whitney test

PFI, Platinum-free interval; SCS, Secondary cytoreductive surgery

**Table 2.** Clinical characteristics of patients assessed for CCNE1 expression.

Characteristic	Frequency (%)
Number of patients	57 (100%)
Age, years^a^ Median (IQR)	56.6 (51.8–63.3)
Stage	
I–II	9 (16.7)
III–IV	45 (83.3)
Tumour histology	
High-grade serous	50 (92.6)
Others	4 (7.4)
Family history of ovarian or breast cancer	
No	32 (59.3)
Yes	22 (40.7)
Residual disease after primary surgery	
≤10 mm	34 (70.8)
>10 mm	14 (29.2)
PFI before bevacizumab – Median (IQR)	10.5 (7.4–14.0)
6–12 months	34 (64.2)
≥12 months	19 (33.3)
CT regimen	
Platinum + taxane	19 (33.3)
Platinum + gemcitabine	34 (59.6)
Platinum + liposomal doxorubicin	3 (5.3)
Monotherapy	1 (1.8)
Number of previous treatment lines	
1	30 (54.5)
2	20 (36.4)
3	3 (5.5)
4	2 (3.6)
Surgery	
Primary cytoreduction	34 (60.7)
Interval cytoreduction	22 (39.3)
SCS	
Yes	22 (38.6)
No	35 (61.4)
CCNE1 overexpression	
Yes	26 (45.6)
No	31 (54.4)
CICE-score	
0	15 (26.3)
1	16 (28.1)
2	10 (17.5)
4	11 (19.3)
6	5 (8.8)

a Continuous variables are described as median and interquartile range

IQR, Interquartile range; PFI, Platinum-free interval

**Table 3.** Univariate analysis of PFS.

Characteristic	HR (95% CI)	*p* value
Age		
<60	1	0.021
≥60	1.68(1.08–2.60)	
Breast or OC positive family history		
No	1	0.983
Yes	1.01 (0.63–1.61)	
Stage		
I–II	1	0.696
III–IV	1.14 (0.58–2.23)	
Histology		
High grade serous	1	0.008
Others	0.14 (0.03–0.61)	
Surgery		
Primary cytoreduction	1	0.409
Interval cytoreduction	1.16 (0.81–1.67)	
Residual disease after primary surgery		
primary surgery		
≤10 mm	1	0.827
>10 mm	1.03 (0.78–1.36)	
PFI before bevacizumab		
6–12 months	1	0.044
≥12 months	0.63 (0.40–0.99)	
Treatment		
CT alone	1	0.225
CT + bevacizumab	0.77 (0.50–1.12)	
Number of previous treatment lines		
1	1	0.264
>1	1.30 (0.82–2.04)	
CCNE1 overexpression		
<2	1	0.069
≥2	1.77 (0.96–3.27)	

PFI, Platinum free interval

**Table 4.** Multivariate analysis for OS and PFS.

	PFS^a^		OS^b^	
Characteristic	HR (95% CI)	*p* value	HR (95% CI)	*p* value
Bevacizumab				
No	1	0.084	1	0.401
Yes	0.54 (0.26–1.09)		0.70 (0.30–1.61)	
Surgery				
Primary cytoreduction	-	-	1	0.088
Interval cytoreduction	-		2.04 (0.90–4.65)	
CCNE1 overexpression				
No	1	0.026	1	0.035
Yes	2.25 (1.10–4.60)		(1.07–5.45)	

a 49 patients with complete data included in the multivariate analysis, 38 events

b 46 patients with complete data included in the multivariate analysis, 36 events

PFS, Progression free survival; OS, Overall survival

**Table 5.** Univariate analysis for OS.

Characteristic	HR (95% CI)	*p* value
Age		
<60	1	0.035
≥60	1.73 (1.04–2.86)	
Breast or OC positive family history		
No	1	0.310
Yes	1.33 (0.77–2.30)	
Stage		
I–II	1	0.157
III–IV	1.95 (0.77–4.92)	
Histology		
High grade serous	1	0.036
Others	0.12 (0.02–0.87)	
Surgery		
Primary cytoreduction	1	0.010
Interval cytoreduction	1.73 (1.14–2.62)	
Residual disease after primary surgery		
primary surgery		
≤10 mm	1	0.234
>10 mm	1.19 (0.89–1.59)	
Treatment		
CT alone	1	0.806
CT + bevacizumab	0.81 (0.47–1.38)	
Number of previous treatment lines		
1	1	0.185
>1	1.42 (0.85–2.37)	
PFI before bevacizumab		
6–12 months	1	0.018
≥12 months	0.52 (0.30–0.91)	
CCNE1 overexpression		
No	1	0.049
Yes	2.07 (1.00–4.28)	

PFI, Platinum free interval
